# Brucellosis awareness and knowledge in communities worldwide: A systematic review and meta-analysis of 79 observational studies

**DOI:** 10.1371/journal.pntd.0007366

**Published:** 2019-05-02

**Authors:** Ning Zhang, Hao Zhou, De-Sheng Huang, Peng Guan

**Affiliations:** 1 Department of Epidemiology, School of Public Health, China Medical University, Shenyang, China; 2 Department of Impression Evidence Examination Technology, Criminal Investigation Police University of China, Shenyang, China; 3 Department of Mathematics, School of Fundamental Sciences, China Medical University, Shenyang, China; Faculty of Science, Ain Shams University (ASU), EGYPT

## Abstract

**Background:**

Brucellosis is regarded as a major zoonotic infection worldwide. Awareness and knowledge of brucellosis among occupational workers is considered an important aspect of brucellosis control in both humans and animals. The aim of this study was to explore the distributions of the pooled awareness level and the knowledge level of the disease worldwide.

**Methods:**

A meta-analysis was carried out to obtain pooled brucellosis awareness levels and knowledge levels of respondents regarding the zoonotic nature of brucellosis, mode of brucellosis transmission, and brucellosis symptoms in animals and humans. The analysis was conducted and reported in accordance with the Preferred Reporting Items for Systematic Review and Meta-analyses guidelines.

**Results:**

A total of seventy-nine original articles reporting the brucellosis awareness levels of in populations from 22 countries were assessed. The total pooled awareness level of brucellosis was 55.5%, and the pooled awareness levels regarding the zoonotic nature of brucellosis, mode of brucellosis transmission, signs of human brucellosis and signs of animal brucellosis were 37.6%, 35.9%, 41.6%, and 28.4% respectively. The pooled awareness level was higher than the brucellosis-related knowledge level. Subgroup analyses showed that no obvious differences in brucellosis awareness levels between high-risk populations in Asia and Africa. Health workers (including human health workers and veterinarians) had the greatest overall awareness and knowledge of human brucellosis. The overall awareness levels and knowledge levels of livestock owners (farmers) and herders were higher than those of dairy farmers and abattoir workers. In addition, awareness and knowledge levels were higher among people who were involved in bovine, caprine and ovine animal production or in caprine and ovine animal production than among people who were involved in only bovine animal production.

**Conclusions:**

Insufficient awareness and knowledge of brucellosis were observed in the original studies conducted mainly in Asia and Africa. Interventions to improve public knowledge about brucellosis are urgently needed.

## Author summary

Brucellosis is considered a neglected zoonotic disease that creates a very large obstacle to the development of animal production and is a great threat to human health. High brucellosis awareness and knowledge is critical for the implementation of correct practices and habits and consequently the control and prevention of brucellosis. The aim of this study was to estimate the awareness and knowledge of brucellosis, specifically regarding its zoonotic nature, mode of transmission, and signs in humans and in animals as well as awareness information sources. To this end, a meta-analysis of data from 79 studies was performed. The included studies on the awareness and knowledge of brucellosis were mainly from Africa and Asia. There were no significant differences in the awareness levels of brucellosis among high-risk groups in Asia and Africa. Overall, people’s awareness and knowledge of brucellosis were low and insufficient. Health workers had the highest pooled levels of awareness and knowledge regarding brucellosis. In addition, livestock stock owners (farmers) had notably higher awareness and knowledge levels than dairy farmers and abattoir workers. Neighbors and friends were the most common sources of brucellosis information for farmers. The low and insufficient awareness and knowledge about brucellosis is an obstacle for public health. Raising awareness and increasing detailed knowledge of brucellosis are of great significance for the control of brucellosis and the protection of human health. The potential of the media and health workers in the dissemination of knowledge about the disease needs to be fostered.

## Introduction

Brucellosis is considered as one of the most important zoonoses in the world with more than 500,000 human cases occurring globally every year [1,2]. Despite a high burden of infection in many areas of the world, brucellosis is rarely prioritized by health systems and is considered a neglected zoonosis by the World Health Organization (WHO) [3] and World Organisation for Animal Health (OIE) [4]. Brucellosis causes abortion, infertility and milk production decline in animals [5,6]. It is transmitted to humans through consumption of unpasteurized dairy products and uncooked meat or through direct contact with infected animals, placentas or aborted fetuses [7]. Clinically, human disease is characterized by fever, fatigue, sweating, joint pain, headache, loss of appetite, muscular pain, lumbar pain, weight loss, and arthritis [8,9] and is often misdiagnosed as other febrile syndromes, such as malaria and typhoid fever, resulting in mistreatments and underreporting [6,10,11].

Generally, poor hygiene, prevalence of the disease in animals and practices that expose humans to infected animals or their products can significantly increase the risk of the occurrence of the disease in humans [12]. Therefore, farmers, pastoralists, abattoir workers, animal health personnel, laboratory personnel and other people involved in the livestock value chain are considered the highest occupational risk groups [13]. Vaccination is an important control tool particularly where there is no compensation for livestock owners for test-and-slaughter, there is no individual identification system and mobile livestock keeping is practiced. And the control and eradication of brucellosis cannot be achieved by vaccination and test-and-slaughter only; the cooperation of relevant occupational groups is an important component in achieving this goal [14]. Therefore, adequate knowledge of the epidemiology of brucellosis is of great public health importance, particularly among high-risk groups, as knowledge promotes people to take protective measures at work and actively participate in disease control programs, thus greatly assisting the development of brucellosis control strategies.

Although there are many original studies that evaluate the knowledge and awareness of brucellosis, the overall awareness and detailed knowledge of the disease and the distribution of the literature remain unclear. To this end, we conducted this meta-analysis study to pool brucellosis awareness and knowledge levels worldwide as well as to seek out factors associated with the levels of awareness and knowledge.

## Materials and methods

### Search strategy

This review was reported in accordance with the Preferred Reporting Items for Systematic Review and Meta-analyses (PRISMA) guidelines [15], and the PRISMA checklist is appended as S1 Appendix. Between March and June 2018, a literature search was conducted in PubMed, Web of Science, China National Knowledge Infrastructure (CNKI), Wan Fang and Yahoo search engines to identify the relevant articles about people’s brucellosis awareness and knowledge globally. The search string applied a combination of related words and was applied to each database separately, using Boolean operators. Searches used in all databases are shown in S2 Appendix. To identify additional relevant citations as much as possible, reference lists of included papers as well as “cited by” and “related information” tools in PubMed were searched. Not only English terms but also corresponding Chinese terms were applied to the Chinese databases.

### Inclusion and exclusion criteria

All primary study designs were considered eligible, thus secondary reports, nonoriginal research, comments, editorials and reviews were directly excluded. Studies were included if they were related to brucellosis awareness or knowledge assessment. Studies conducted to evaluate the awareness and knowledge levels of zoonotic diseases were included as long as they reported data about brucellosis, but only data related to brucellosis were considered and analyzed.

Studies containing any of the following criteria were included: (i) studies reporting the awareness of brucellosis, where the original expression was similar to “Have you heard of brucellosis?”, “Do you know about brucellosis?” or “be aware of brucellosis”; (ii) studies reporting brucellosis knowledge about the mode of transmission to people, the zoonotic nature, and signs in humans and animals; (iii) studies reporting knowledge about consumption of unpasteurized milk and uncooked meat as high-risk practices for brucellosis infection in humans; and (iv) studies providing the information sources of people who had heard of brucellosis.

### Screening of the identified publications

All citations were imported and duplicates were removed using the software EndNote X8. Two team members independently screened the literature in two stages. In the first stage, titles and abstracts were screened to exclude duplicates and ineligible studies based on relevance. In the second stage, the two reviewers independently evaluated the full text of the selected literature to ensure full compliance with the inclusion criteria. At each stage, the selected papers were compared by the two investigators for analysis consistency. At the event of a disagreement, a third investigator joined the discussion and made a decision. The screening and selection of studies were promoted by the creation of appropriately labeled subgroups in EndNote.

### Data extraction

A data abstraction form was constructed after screening the selected articles. For each included study, we extracted the following basic information: author, publication year, geographic region, study design, study population, sampling method, number of participants, education distribution, gender distribution and main livestock contacted by the studied population. Furthermore, the number of participants who answered positively (n) and sample size (N) were the two necessary parameters for the calculation of the pooled levels of brucellosis awareness and knowledge in the meta-analysis. In particular, the number of participants who answered positively (n) was obtained directly from these studies or by multiplying the sample sizes (N) with the proportions (%) associated with the investigated items reported in the studies. All the data extraction work was performed independently and then compared by two investigators. In the event of a disagreement, a third person joined the discussion and made a decision.

### Data analyses

All available data were pooled in the present meta-analysis. The subgroups and categories considered included geographic regions (classified into five regions, Asia, Africa, South/Central America, North America and Oceania), animal species (bovine, ovine and caprine), human populations (occupational and nonoccupational groups; farmers, abattoir workers, traders, human and animal health workers, pastoralists and livestock transporters were identified as the occupationally exposed population) and countries. Additional subgroup analyses were performed for specified occupations (animal and human health workers, livestock owners (farmers), dairy farmers, abattoir workers, pastoralists, patients, students and residents).

Meta-analysis was performed based on a random-effect model. To stabilize the variance, the original rates were transformed by arcsine transformation. Cochran’s chi-square (Q-test) and the *I*-square (*I*^*2*^) statistic were used to estimate the heterogeneity of the results. A funnel plot was constructed to visually examine the publication bias, and Begger’s rank test was used to test the significance of the plot’s asymmetry. R statistical software (Version 3.0.0) was applied for all the aforementioned calculations.

### Risk of bias assessment

The quality and risk of bias of studies were assessed comprehensively as outlined in Hoy et al. [16] and Crombie et al. [17]. The risk of bias in the included studies was evaluated with a total of ten risk-biased items regarding external validity (items 1 to 4 assessed domain selection and nonresponse bias) and internal validity (items 5 to 9 assessed the domain of measurement bias, and item 10 assessed the bias related to the analysis). For each item, the study was classified as “Yes” or “No”, which meant “Low risk” or “High risk”, respectively. At the end of the overall risk assessment of study bias, studies with a “No” score ≤3 were classified as low risk, studies with a “No” score 4–6 were classified as moderate risk and studies with a “No” score ≥7 were classified as high risk. The risk bias and assessment results are provided in S3 Appendix. Studies with overall high risk of study bias were still included in this present meta-analysis as long as the research purpose and design were reasonable and the numerator and denominator for the parameter of interest were appropriate.

## Results

### Characteristics of the included studies

**Fig 1:**
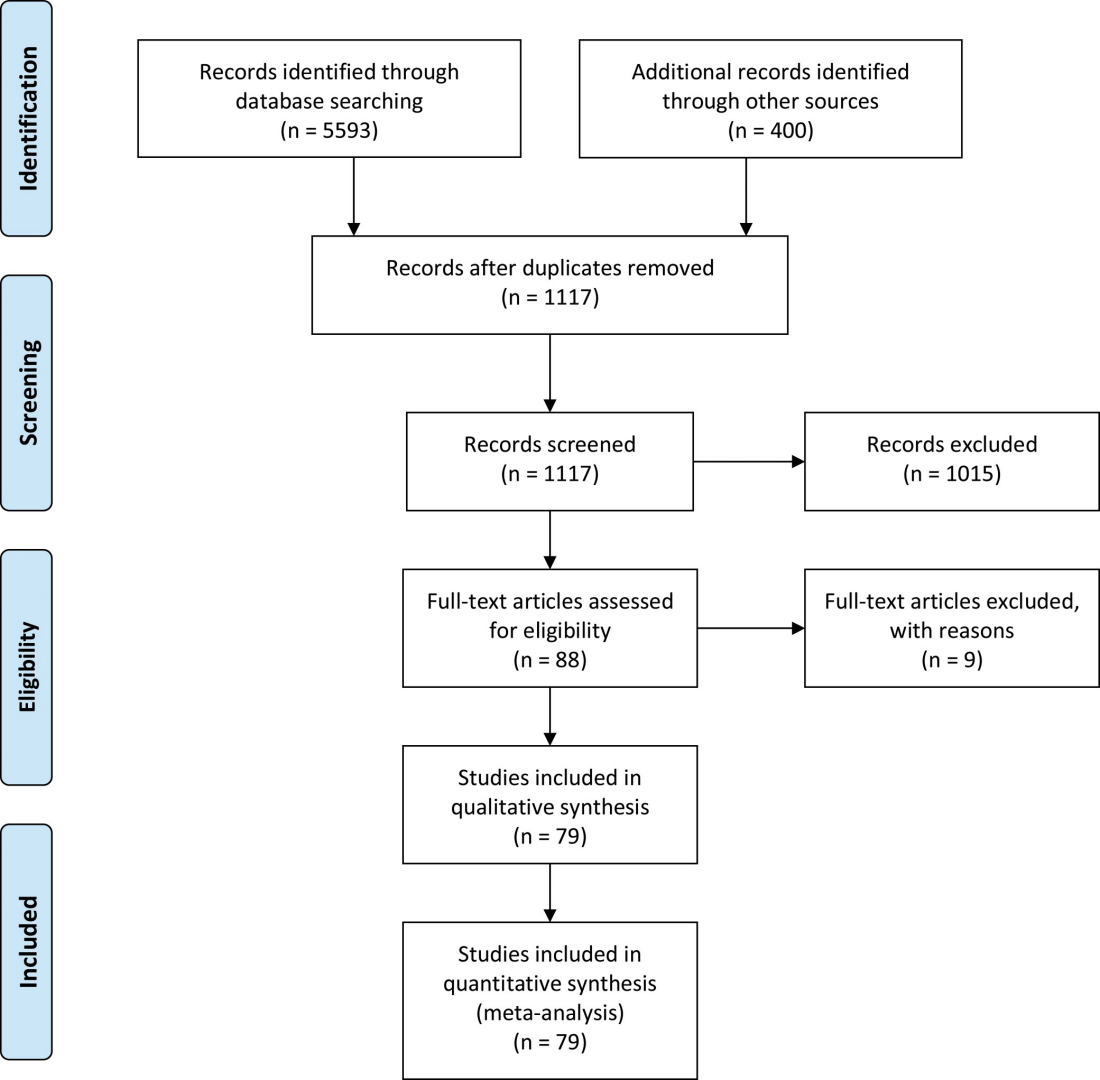
The Preferred Reporting Items for Systematic Reviews and Meta-Analyses flow diagram.

The search and selection process of related studies is presented in Fig 1. After the removal of articles published before 2010, articles with data that could not be interpreted, articles with duplicated data and studies without full-text, seventy-nine studies were included in the meta-analysis.

The characteristics of the included studies are provided in Table 1. Among the included publications, 52 studies were from Asia, 24 were from Africa, one each from Europe, South/Central America and North America, respectively. Among the included studies, one was published in Portuguese, one was published in Turkish, 31 were published in Chinese, and 56 were published in English.

**Table 1:** Characteristics of the included studies regarding the human brucellosis awareness in the meta-analysis.

Geographical region	First author, Publication year	Country	Investigation time	Questionnaire administration	Characteristics of participants				Illiterate level (%)	Main animal	Sampling method
					Occupation	Sample size	Age (yrs, mean, range)	Female (%)			
Africa	Mosalagae, 2010 [18]	Zimbabwe	Oct, 2009-Mar, 2010	Interviewed	Dairy farmers	119	˗	25.2	28.6	Cattle	Convenience
	Holt, 2011 [19]	Egypt	Dec, 2009-Feb, 2010	Interviewed	Livestock owners	214	˗	50.0	˗	Cattle and buffaloes	Random
	Mufinda, 2011 [20]	Angola	Nov, 2009	Interviewed	Breeders and abattoir workers	170	˗	7.3	˗	Cattle, goats, sheep, pigs	Random
	Adesokan, 2013 [21]	Nigeria	-	Interviewed	Livestock owner, traders	157	41.7 (18–70)	16.6	˗	Cattle	Cluster
	Chikerema, 2013 [22]	Zimbabwe	Feb-Nov, 2010	Interviewed	Livestock owners	326	˗	˗	˗	Cattle	Random
	Tesfaye, 2013 [23]	Ethiopia	Nov, 2011-Apr, 2012	Interviewed	High-risk population	175	15–64+	29.0	18.4	Livestock	Random
	Kansiime, 2014 [24]	Uganda	Jun-Aug, 2012	Interviewed	Pastoralists	371	40 (18–60+)	51.0	˗	Cattle	Random
	Tebug, 2014 [25]	Malawi	Feb, 2011–Jun, 2011	Interviewed	Dairy farmers	140	˗	60.0	71.4	Cattle	Random
	Bashaka, 2015 [26]	Tanzania	Nov, 2013-Sep, 2014	Interviewed	Farmers, food vendors	260	˗	100.0	64.2	Cattle, sheep and goat	Random
	Buhari, 2015 [27]	Nigeria	-	Interviewed	Pastoralists	42	˗	˗	˗	Cattle	Random
	Desta, 2015 [28]	Ethiopia	-	Interviewed	Farmers, human and animal health workers	320			79.2	Camel	Random and Convenience
	Mufinda, 2015 [29]	Angola	-	Interviewed	Abattoir workers and Breeders	323	36.2 (16–71)	35.1	58.5	Cattle	Random
	Obonyo, 2015 [30]	Kenya	Oct-Nov, 2013	Interviewed	Pastoralists	120	15–70	25.0	77.0	Sheep and goat	Random
	Tebug, 2015 [31]	Senegal	Aug-Nov, 2013	Interviewed	Livestock owners	222	16–85	15.8	57.7	Cattle	Random
	Abera, 2016 [32]	Ethiopia	-	Interviewed	Livestock owners	500	˗	˗	˗	Livestock	Random
	Hegazy, 2016 [33]	Egypt	Feb-Jul, 2014	Interviewed	Pastoralists	26	˗	˗	˗	Sheep and goat	Census
	Zhang, 2016 [34]	Tanzania	-	Interviewed	Human and animal healthcare provider	62	23–81	˗	˗	˗	Census
	Eldeihy, 2017 [35]	Egypt	-	Interviewed	Livestock owners	69	˗	˗	˗	Cattle,Buffalo, sheep, goat	˗
	Madut, 2017 [36]	Susan	-	Interviewed	Abattoir workers and patients	650	˗	˗	˗	˗	Purposive
	Marin, 2017 [37]	Susan	Dec, 2015-Jan, 2016	Interviewed	Abattoir workers and animal health Worker	77	29.9 (15–58)	3.1	53.2	˗	˗
	Nabirye, 2017 [38]	Uganda	Mar, 2014-Feb, 2015	Interviewed	Patients	251	10–84	53.0	60.5	˗	Convenience
	Njuguna, 2017 [39]	Kenya	Dec 2015-May 2016	Interviewed	Cattle owners	80	19–60+	70.0	24.0	Cattle	Random
	Wakene, 2017 [40]	Ethiopia	Oct, 2016-Apr, 2017	Interviewed	Pastoralist and human health personnel	126	˗	˗	˗	Sheep and goat	Random
	Nyokabi, 2018 [41]	Kenya	-	Interviewed	High-risk population	154	˗	-	˗	Cattle, camel, sheep, goat	Purposive, snowball
Asia	Chen, 2010 [42]	China	Jan, 2007-Dec, 2009	Interviewed	High-risk population	916	41 (35–50)	28.3	˗	Cattle, sheep and goat	Random
	FAO, 2010 [43]	Tajikistan	-	Interviewed	Livestock owners	500	˗	˗	˗	Cattle, sheep and goat	˗
	Hou, 2010 [44]	China	2009	Interviewed	Herdsmen	217	˗	˗	˗	Cattle, sheep and goat	Census
	Jini, 2010 [45]	China	Jul, 2008	Interviewed	Farmers	563	>15	46.9	11.2	Cattle, sheep and goat	Random
	Akkus, 2011 [46]	Turkey	May-Jun, 2010	Interviewed	Breeder	97	44.3	50.0	34.0	Cattle, sheep and goat	˗
	Guo, 2011 [47]	China	-	Interviewed	High-risk population	300	18–60	28.3	5.3	Cattle, sheep and goat	Random
	Zhou, 2011 [48]	China	-	Interviewed	Traders	160	16–87	51.2	10.0	Sheep and goat	Census
	Mohan, 2012 [49]	India	-	Interviewed	Dairy farmers	240	˗	33.8	16.0	Cattle and buffaloes	Random
	Qi, 2012 [50]	China	-	Interviewed	Residents	99	45.5 (18–69)	58.6	24.2	˗	Convenience
	Grahn, 2013 [51]	Tajikistan	Apr, 2011	Interviewed	Livestock owners	97	˗	40.0	˗	Sheep and goat	Random
	Huo, 2013 [52]	China	Five weeks in the autumn of 2012	Interviewed	Herdsmen	1538	>15	48.0	˗	Cattle, sheep and goat	Random
	Li, 2013 [53]	China	Nov-Dec, 2008	Interviewed	Breeders	595	33.8 (5–60+)	43.9	˗	Sheep and goat	Random
	Liu, 2013 [54]	China	-	Interviewed	High-risk population	144	˗	˗	˗	Cattle, sheep and goat	˗
	Lv, 2013 [55]	China	2012	Interviewed	High-risk population	244	55 (19–88)	44.7	33.2	Cattle, sheep and goat	Random
	Yong, 2013 [56]	China	Jul, 2012	Self-administered	Human health workers	75	42.8 (22–60)	50.0	˗	˗	Random
	Adraiti, 2014 [57]	China	Jun, 2012	Interviewed	Farmers	1200	7–60	˗	˗	Cattle, sheep and goat	Random
	Guan, 2014 [58]	China	Jul, 2013	Interviewed	Students	206	13 (5–19)	46.0	˗	˗	Cluster
	Yang, 2014 [59]	China	Nov, 2012	Interviewed	High-risk population	147	50.6 (20–79)	37.0	˗	Sheep and goat	Census
	Çakmur, 2015 [60]	Turkey	May, 2013	Interviewed	Farmers and Livestock farmers	151	41.7 (14–86)	45.0	19.9	Cattle, sheep and goat	Convenience
	Li, 2015 [61]	China	Jun-Oct, 2013	Interviewed	High-risk population	257	˗	˗	˗	Cattle, sheep and goat	˗
	Lindahl, 2015 [62]	Tajikistan	-	Interviewed	Dairy farmers	441	˗	78.0	0.7	Cattle	Random
	Musallam, 2015 [63]	Jordan	May-Oct, 2011	Interviewed	Livestock owners	537	˗	˗	˗	Cattle, sheep and goat	Random
	Tong, 2015 [64]	China	May-Oct, 2013	Interviewed	High-risk population	41	48.0 (26–62)	14.6	˗	Sheep and goat	Census
	Zong, 2015 [65]	China	Oct, 2014	Interviewed	High-risk population	160	19–81	33.2	33.6	Cattle, sheep and goat	Random
	Chang, 2016 [66]	China	2011	Self-administered	Students	300	˗	52.	˗	˗	Cluster
	Cheng, 2016 [67]	China	-	Interviewed	High-risk population	493	˗	34.5	6.7	Cattle, sheep and goat	Random
	Deka, 2016 [68]	India	-	Interviewed	Dairy Farmers	292	˗	˗	˗	Cattle	-
	Hundal, 2016 [69]	India	-	Interviewed	Livestock owners	250	˗	˗	˗	Livestock	Random
	Kolhe, 2016 [70]	India	Aug, 2015	Interviewed	Women(residents)	300	˗	100.0	1.7	˗	Random
	Li, 2016 [71]	China	-	Interviewed	Breeders	802	55.7 (23–83)	24.9	22.9	Sheep and goat	Random
	Shao, 2011 [72]	China	-	Interviewed	Workers in Livestock marketers	199	16–87	48.7	˗	Cattle, sheep and goat	Census
	Parahakar, 2016 [73]	India	Feb-Mar, 2015	Interviewed	Butchers	86	˗	8.1	12.8	Livestock	Random
	Peng, 2016 [74]	China	-	Interviewed	High-risk population	308	>15	29.5	˗	Sheep and goat	Census
	Rajkumar, 2016 [75]	India	-	Interviewed	Livestock owners	250	˗	˗	˗	Livestock	Random
	Rajput, 2016 [76]	India		Interviewed	Dairy farmers	120	˗	˗	˗	Cattle and buffaloes	Random
	Tian, 2016 [77]	China	-	Interviewed	Residents	2491	21–60	38.7	61.6	Cattle, sheep and goat	Random
	Zhang, 2016 [78]	China	-	Interviewed	Breeders	191	25–79	32.5	˗	Sheep and goat	Random
	Zhu, 2016 [79]	China	2014	Interviewed	Dairy farmers	81	19–66	44.4	˗	Cattle	Random
	Arif, 2017 [80]	Pakistan	Feb-Jun, 2015	Interviewed	Dairy farmers	420	˗	64.0	46.0	Cattle and buffaloes	Random
	Awwad, 2017 [81]	Palestine	2013–2014	Self-administered	Livestock owners	118	˗	20.3	6.8	Sheep and goat	Cluster
	Kant, 2017 [82]	India	-	Interviewed	Livestock owners	100	˗	˗	˗	Cattle	˗
	Li, 2017 [83]	China	-	Interviewed	High-risk population	200	47.4 (19–80)	28.0	˗	Sheep and goat	Cluster
	Liu, 2017 [84]	China	Nov, 2016	Self-administered	Human health workers	819	˗	66.1	˗	˗	Census
	Mangalgi, 2017 [85]	India	-	Interviewed	Veterinarians	1084	39.8 (20–60)	˗	˗	˗	Cluster
	Munisamy, 2017 [86]	India	-	Interviewed	Dairy farmers	100	˗	27.0	75.0	Cattle	-
	Singh, 2017 [87]	India	-	Interviewed	Butchers	100	18–50+	4.0	81.0	Livestock	Random
	Xiao, 2017 [88]	China	-	Interviewed	High-risk population	178	48.0 (15–72)	21.9	˗	Cattle, sheep and goat	Cluster
	Yuan, 2017 [89]	China	-	Interviewed	Breeders	403	56.5 (26–88)	38.5	13.6	Sheep and goat	Random
	Zhang, 2017 [90]	China	Nov, 2012	Interviewed	Breeders	403	44.1	42.9	˗	Sheep and goat	Random
	Gao, 2018 [91]	China	Feb, 2014	Interviewed	High-risk population	265	15–78	38.4	˗	Cattle, sheep and goat	Cluster
	Kothalawala, 2018 [92]	Sri Lanka	Aug-Sep, 2016	Interviewed	Dairy farmers	155	˗	19.9	˗	Cattle	Random
	Zeng, 2018 [93]	China	Apr-Aug, 2015	Interviewed	Pastoralists	317	50.1 (20–80)	18.3	33.4	Cattle	Random
Europe	Diez, 2013 [94]	Portugal	Apr-July, 2012	Interviewed	Cattle Farmers	154	˗	14.3	˗	Cattle	Census
North America	Crow, 2013 [95]	America	Jul, 2012-Sep, 2012	Self-administrated	Dog Breeders	75	56 (26–80)	78.7	˗	Dog	Census
South America	Ruano, 2017 [96]	Ecuador	-	Interviewed	High-risk population	500	˗	32.2	7.7	Cattle	Random

The target populations of the studies included human health workers, high-risk occupational populations (farmers, traders, abattoir workers, livestock transporters, and animal health workers.), students and residents. Main animal species reared by the respondents were cattle and buffalo, sheep and goats, pigs, camels and dogs. The sample sizes of the studies ranged from 26 to 2,491 respondents. A questionnaire-based survey was administered in all the included studies; five studies adopted a self-administered questionnaire, while 74 studies collected the data during face to face interviews.

### Risk of bias assessment result

A low risk of bias was found in 63 studies, a moderate risk of bias was found in 15 studies and a high risk of bias was indicated in one study, which was included due to its reasonable research purpose and study design. The detailed risk of bias of each study is shown in S3 Appendix. In addition, with Begger's test, no evidence of publication bias was found (Table 2).

**Table 2:** The pooled awareness and knowledge levels of brucellosis.

Studied items	Number of studies	Level (95%CI)	*I*^*2*^(%)	*P*-value	Begger’s test (*P*-value)
Heard of (aware of) brucellosis	52	55.5 (45.4, 65.4)	99.4%	<0.0001	0.85
Zoonotic nature of brucellosis	33	37.6 (25.7, 50.4)	99.4%	<0.0001	0.76
Mode of transmission	30	35.9 (25.3, 47.3)	99.0%	<0.0001	0.97
Clinical signs of human brucellosis	23	41.6 (33.0, 50.4)	98.8%	<0.0001	0.25
Fever	17	34.4 (19.5, 51.1)	98.9%	<0.0001	0.43
Fatigue	10	30.7 (12.6, 52.6)	99.1%	<0.0001	0.33
Joint pain	17	32.1 (21.2, 44.1)	98.2%	<0.0001	0.41
Sweating	11	21.8 (12.5, 32.9)	97.0%	<0.0001	0.94
Urogenital diseases	6	9.3 (1.9, 21.5)	96.5%	<0.0001	0.85
Symptoms of animal brucellosis	16	28.4 (21.9, 35.5)	97.4%	<0.0001	0.69
Abortion	16	37.2 (23.7, 51.8)	98.5%	<0.0001	0.75
Reduction in milk production	5	18.5 (4.0, 40.2)	97.8%	<0.0001	1
Animal source for brucellosis infection					
Sheep and goat	9	54.1 (47.3, 60.8)	92.1%	<0.0001	0.53
Cattle	9	29.1 (17.4, 42.5)	97.6%	<0.0001	1
Pig	8	17.5 (10.3, 26.2)	95.6%	<0.0001	0.22
Dog	7	12.8 (7.0, 20.0)	94.8%	<0.0001	0.88
High-risk practices for infection					
Consumption of raw milk	21	44.5 (30.0, 59.4)	99.2%	<0.0001	0.67
Consumption of raw meat	19	34.6 (23.2, 47.1)	98.9%	<0.0001	0.28
Direct contact with aborted fetuses and abortion material	14	54.9 (37.0, 72.1)	99.4%	<0.0001	0.78
Vaccination as a preventive measure of brucellosis	15	26.1 (12.1, 43.3)	99.4%	<0.0001	0.07
Information sources of awareness of brucellosis					
Neighbor relative or friends	9	58.7 (31.9, 82.9)	99.3%	<0.0001	1
TV and radio	9	23.1 (8.4, 42.4)	98.2%	<0.0001	0.40
Local health workers	7	17.8 (9.7, 27.6)	93.4%	<0.0001	0.76
Lecture	5	7.9 (3.6, 13.6)	87.0%	<0.0001	0.33

### Awareness of brucellosis, its zoonotic nature and its transmission mode

An awareness of brucellosis was reported in 52 studies, with a pooled awareness level of 55.5%. An awareness of the zoonotic nature of brucellosis and its transmission mode were reported in 33 and 30 studies, respectively, with respective pooled awareness levels of 37.6% and 35.9%, as shown in Table 2.

### Awareness of the symptoms of brucellosis in humans and animals

An awareness of the clinical signs and symptoms of human brucellosis and animal brucellosis were reported in 23 and 16 studies, respectively, and the pooled awareness levels were 41.6% and 28.4%, respectively. In addition, we explored the distribution of brucellosis symptoms that were mentioned in the included studies. Fever, fatigue, joint pain, sweating and urogenital disease were the most commonly mentioned and studied symptoms in humans, but the pooled awareness level was lower than 35.0%. Abortion was the most commonly mentioned symptom of animal brucellosis, with a pooled awareness level of 37.2%, followed by a reduction in milk production (18.5%), as shown in Table 2.

### Awareness of zoonotic infection and high-risk practices for human infection

Nine included studies explored the awareness of infected animals as the source of human infection, with a pooled awareness level of 54.1%; respondents listed sheep and goats as an animal source, followed by cattle, pigs and dogs as an infection source. The pooled awareness levels of raw milk consumption and the consumption of infected meat as risk factors for brucellosis were 44.5% and 34.6%, respectively. The pooled knowledge level of direct contact with aborted fetuses and abortion materials as high-risk practice was 54.9% (Table 2).

### Awareness regarding the vaccination and brucellosis information sources

Fifteen studies explored the awareness regarding the vaccination of animals against brucellosis, and the pooled awareness was only 26.1% (Table 2). Nine studies analyzed the information sources of those respondents who had heard of brucellosis. People mainly acquired knowledge of brucellosis from the following four sources: neighbors/friends, mass media (TV/radio), health workers and health education-related lectures. Overall, 58.7% of respondents acquired the information about brucellosis through their neighbors or friends, which was notably higher than those that acquired information through TV/radio, health workers and lectures (Table 2).

### Subgroup analyses by occupation, animal species and geographic region

Regarding the awareness of brucellosis, no obvious differences were found between the occupation-related population and students and residents. Subgroup analysis by occupation showed that animal health workers had the greatest awareness of brucellosis (100.0%). Pastoralists had higher awareness of brucellosis (72.0%) than livestock owners/farmers (57.0%), abattoir workers (24.3%), dairy farmers (29.5%) and livestock (product) traders (30.3%). We also found that people who were involved in bovine, ovine and caprine production (72.5%) and ovine and caprine production (74.3%) had higher awareness levels than those people who were involved in only bovine production (35.6%), as shown in Tables 3 and 4.

**Table 3:** Subgroup analysis of awareness and knowledge of brucellosis.

Items	Subgroups	Population	Number of studies	Level (95%CI)	*I*^*2*^	*P*-Value
Heard of brucellosis (aware of brucellosis)	Population	Occupational population	48	55.2 (44.4, 65.8)	99.4%	<0.0001
		Resident	1	78.8	-	-
		Student	2	45.5 (35.2, 55.9)	95.9%	0.02
	Animal	Bovine	20	35.6 (19.2, 54.0)	99.5%	<0.0001
		Bovine, caprine and ovine	15	72.5 (52.3, 88.8)	99.6%	<0.0001
		Caprine and ovine	9	74.3 (58.7, 87.2)	98.8%	<0.0001
		Dog	1	88.0	-	-
		Camel	1	7.7	-	-
	Region	Africa	20	53.4 (36.3, 70.2)	99.2%	<0.0001
		Asia	30	56.5 (43.0, 69.5)	99.5%	<0.0001
		North America	1	88.0	-	-
		South America	1	30.2	-	-
Zoonotic disease	Population	Occupational population	32	39.4 (27.5, 52.0)	99.3%	<0.0001
		Resident	1	0.7 (0.1, 1.9)	-	-
	Animal	Bovine	10	21.2 (6.2, 42.0)	99.2%	<0.0001
		Bovine, caprine and ovine	8	54.7 (35.3, 73.4)	99.7%	<0.0001
		Caprine and ovine	9	62.2 (53.5, 70.5)	93.2%	<0.0001
		Dog	1	58.7	-	-
	Region	Africa	9	17.8 (2.7, 42.1)	99.2%	<0.0001
		Asia	22	44.0 (30.8, 57.6)	99.3%	<0.0001
		Europe	1	74.7	-	-
		South America	1	58.7	-	-
Mode of transmission	Population	Occupational population	17	37.4 (27.0, 48.5)	99.0%	<0.0001
		Resident	1	13.3	-	-
	Animal	Bovine	4	26.4 (16.8, 37.4)	95.8%	<0.0001
		Bovine, caprine and ovine	8	43.2 (23.4, 64.2)	99.3%	<0.0001
		Caprine and ovine	5	28.3 (12.2, 47.9)	99.2%	<0.0001
	Region	Africa	6	45.1 (30.2, 60.4)	96.5%	<0.0001
		Asia	11	32.0 (18.2, 47.7)	99.5%	<0.0001
		South America	1	26.0	-	-
Symptoms of human	Population	Occupational Population	22	41.6 (32.7, 50.8)	98.9%	<0.0001
		Student	1	40.0	-	-
	Animal	Bovine	4	14.8 (2.8, 33.8)	98.7%	<0.0001
		Bovine, Caprine and ovine	10	46.6 (35.2, 58.2)	98.4%	<0.0001
		Caprine and ovine	6	46.2 (33.8, 58.8)	96.4%	<0.0001
	Region	Africa	2	18.7 (0.0, 58.7)	99.0%	-
		Asia	20	45.1 (36.1, 54.1)	98.7%	<0.0001
		South America	1	23.4	-	-
Symptoms of animals	Population	Occupational Population	15	29.4 (22.6, 36.8)	97.5%	<0.0001
		student	1	15.1	15.0%	-
	Animal	Bovine	5	28.9 (22.6, 35.6)	90.5%	<0.0001
		Bovine, Caprine and ovine	6	31.3 (21.1, 42.4)	97.3%	<0.0001
		Caprine and ovine	4	27.4 (13.9, 43.6)	97.3%	<0.0001
	Region	Africa	3	30.4 (19.2, 42.9)	94.4%	<0.0001
		Asia	12	27.9 (19.5, 37.3)	98.0%	<0.0001
		South America	1	29.8	-	-
Vaccination as a preventive measure for brucellosis	Population	Occupational Population	14	26.1 (11.3, 44.5)	99.4%	<0.0001
		student	1	26.0		
	Animal	Bovine	3	44.9 (1.0, 95.8)	99.8%	<0.0001
		Bovine, Caprine and ovine	7	26.4 (10.9, 45.7)	99.0%	<0.0001
		Caprine and ovine	1	5.0		
	Region	Africa	6	4.6 (0.6, 12.2)	93.5%	<0.0001
		Asia	9	46.3 (27.8, 65.4)	99.3%	<0.0001

**Table 4:** Subgroup analyses of awareness and knowledge among occupations.

Items	Occupations	Number of studies	Level (95%CI)	*I*^2^	*P*-Value
Heard of brucellosis (aware of brucellosis)	Abattoir worker	7	24.3 (15.2, 34.8)	81.1%	<0.0001
	Dairy farmer	8	29.5 (11.4, 51.8)	99.0%	<0.0001
	Animal health worker	3	100.0 (98.6, 100.0)	0%	1
	Human health worker	3	78.6 (7.29, 100.0)	98.8%	<0.0001
	Livestock (product) trader	3	30.3 (24.9, 36.0)	0.0%	0.4950
	Livestock owner (farmer)	14	57.0 (39.1, 74.0)	99.6%	<0.0001
	Pastoralist	5	72.0 (30.5, 98.3)	99.4%	0.0010
	Brucellosis patient	3	55.1 (45.4, 64.7)	78.9%	0.0087
	Resident	1	78.8	-	-
	Transporter	1	71.4	-	-
	Student	2	45.5 (35.2, 55.9)	82.1%	0.0180
Zoonotic disease	Abattoir worker	3	2.6 (0.0, 11.2)	87.2%	<0.0001
	Dairy farmer	8	15.4 (2.1, 37.8)	99.5%	<0.0001
	Livestock owner (farmer)	10	59.9 (38.2, 79.7)	99.1%	<0.0001
	Pastoralist	3	34.8 (17.3, 54.7)	93.2%	0.0004
	Resident	1	0.7	-	-
Mode of transmission	Abattoir worker	3	2.4 (0.0, 20.3)	93.3%	<0.0001
	Dairy farmer	2	7.4 (0.7, 20.5)	97.0%	<0.0001
	Animal health worker	2	75.9 (0.4, 100.0)	96.2%	<0.0001
	Human health worker	2	80.9 (58.2, 96.0)	92.2%	0.0003
	Livestock (product) trader	1	39.8	-	-
	Livestock owner (farmer)	6	27.2 (16.7, 39.2)	97.2%	<0.0001
	Patient	2	30.1 (1.0, 76.1)	96.1%	<0.0001
	Resident	1	13.3	-	-
Human brucellosis symptoms	Abattoir worker	2	18.3 (3.5, 41.2)	79.6%	0.0270
	Dairy farmer	1	3.1	-	-
	Animal health worker	2	50.5 (45.5, 55.5)	5.9%	0.3025
	Human health worker	1	75.8	-	-
	Livestock (product) trader	1	7.8	-	-
	Livestock owner (farmer)	7	31.9 (19.2, 46.1)	98.2%	<0.0001
	Pastoralist	2	74.3 (72.2, 76.3)	88.8%	0.7530
	Patient	2	48.1 (34.3, 62.1)	60.7%	0.1107
	Student	1	40.0	-	-
Animal brucellosis symptoms	Patient	1	4.3	96.4%	<0.0001
	Student	1	37.9	-	-
	Livestock owner (farmer)	6	26.4 (13.6, 41.5)	98.8%	<0.0001
	Pastoralist	1	19.4	-	-
	Patient	1	53.1	-	-
	Student	1	15.1	-	-
Vaccination as a preventive measure	Abattoir worker	2	9.5 (1.1, 25.1)	82.5%	0.0168
	Dairy farmer	1	88.4		
	Animal health worker	1	30.0		
	Human health worker	1	1.9		
	Livestock owner (farmer)	7	19.3 (1.9, 48.5)	99.6%	<0.0001
	Pastoralist	2	25.9 (0.0, 82.3)	99.2%	<0.0001

Regarding the zoonotic nature of brucellosis, people involved mainly in bovine, ovine and caprine production had an awareness level of 54.7% and people involved in ovine and caprine had an awareness level of 62.2%, while people involved in only bovine production had an awareness level of 21.2%. The pooled awareness level of the zoonotic nature of brucellosis in the African population (17.8%) was notably lower than that in the Asian population (44.0%). The results indicated that there was no clear difference in the brucellosis awareness levels between Asia (56.5%) and Africa (53.4%) (Table 3). Livestock owners (farmers) showed relatively higher awareness of the zoonotic nature of brucellosis than dairy farmers (15.4%) and abattoir workers (2.6%) (Table 4).

Regarding the mode of transmission from infected animal to human, a low awareness level (37.4%) was found in the occupationally exposed population, whereas a relatively higher awareness level was found in human health care providers (80.9%) and animal health workers (75.9%). Abattoir workers and dairy farmers had extremely low awareness levels (Tables 3 and 4).

Regarding awareness of the symptoms of human brucellosis, higher awareness levels were found in human health care providers (75.8%), animal health workers (50.5%) and pastoralists (74.3%) than in abattoir workers (18.3%) and dairy farmers (3.1%). The awareness among people involved in bovine, ovine and caprine production (46.6%) and ovine and caprine production (46.2%) were notably higher than people involved in only ovine production (14.8%). Regarding regions, the awareness of human brucellosis symptoms was higher in Asia (45.1%) than in Africa (18.7%). An extremely low awareness level of animal symptoms was observed, and no obvious differences were found among geographic regions and people involved in different animal production methods. (Tables 3 and 4).

Regarding the awareness of vaccination of animals against brucellosis, the pooled awareness level in the African population (4.6%) was notably lower than that in the Asian population (46.3%) (Table 3). And the high awareness level of vaccination as a preventive measure for brucellosis was only found in dairy farmers (88.4%) (Table 4).

For the awareness level of brucellosis among the high-risk population (animal health workers, farmers, abattoir workers, traders and transporters other related populations, not including human health workers), no significant difference (*P* = 0.8) was observed between Asia and Africa. The results showed extremely low awareness of brucellosis in India (13.7%), Sri Lanka (11.6%), Angola (23.9%), Ethiopia (17.3%), Zimbabwe (21.0%) and Senegal (0.0%) (Table 5).

**Table 5:** Brucellosis awareness of high-risk populations in countries in Asia and Africa.

Geographic regions	Country	Number of studies	Level (95%CI)	*I*^*2*^	*P*-Value
Overall		47	55.3 (44.3, 66.0)	99.4%	<0.0001
Between Asia and Africa					0.822
Asia		27	56.4 (41.8, 69.9)	99.5%	<0.0001
	China	13	63.0 (45.6, 78.8)	99.5%	<0.0001
	India	5	13.7 (0.4, 40.7)	98.7%	<0.0001
	Tajikistan	3	53.6 (5.6, 97.2)	99.7%	<0.0001
	Turkey	2	78.2 (53.5, 95.2)	94.2%	<0.0001
	Jordan	1	100.0	_	_
	Pakistan	1	70.0	_	_
	Palestine	1	100.0	_	_
	Sri Lanka	1	11.6	_	_
Africa		20	53.9 (36.5, 70.6)	99.2%	<0.0001
	Angola	2	23.9 (3.4, 55.2)	98.0%	<0.0001
	Egypt	2	77.1 (62.6, 88.8)	77.2%	0.0361
	Ethiopia	3	17.3 (8.7, 28.2)	98.7%	<0.0001
	Kenya	3	72.8 (54.2, 88.0)	92.6%	<0.0001
	Nigeria	2	63.2 (30.1, 100.0)	98.7%	<0.0001
	Susan	2	48.8 (26.8, 71.1)	93.4%	<0.0001
	Tanzania	2	95.1 (68.1, 100.0)	82.6%	0.0164
	Uganda	2	88.2 (35.1, 100.0)	99.5%	<0.0001
	Zimbabwe	1	21.0	_	_
	Senegal	1	0.0	_	_

## Discussion

Raising the awareness of brucellosis and brucellosis-related knowledge in occupation-related groups is an important aspect for the effective control of brucellosis [97]. Health education about the disease for high-risk groups was essential in gaining support for a control program [98,99]. Therefore, assessing the overall disease awareness level of the occupational population is a basis for the development and implementation of more efficient health education activities and brucellosis control programs that should fit the needs and perceptions of local communities [100].

This is the first systematic review and meta-analysis aimed at exploring the brucellosis awareness level worldwide. Most of the original studies that assessed the awareness and knowledge of brucellosis were conducted in Asia and Africa, and with less from Europe, America and Oceania, which is generally consistent with the geographical distribution of brucellosis. Brucellosis is endemic to Asia and Africa, and countries in central and southwestern Asia are currently seeing the greatest increase in cases [101,102].

Overall, only approximately half of the occupation-related groups knew about brucellosis, which means that awareness and knowledge of brucellosis were insufficient. The knowledge levels regarding the zoonotic nature, mode of transmission and symptoms in humans and animals of brucellosis were lower than the awareness level of brucellosis, which means that people had heard of brucellosis but did not necessarily have a clear understanding of brucellosis. This might suggest that people in Asia and Africa have superficial and inadequate knowledge about brucellosis. Poor knowledge about brucellosis is an obstacle for brucellosis control and elimination [103]. The low awareness and knowledge levels elucidated in this study are therefore of great importance, particularly considering the zoonotic nature and the public health significance of brucellosis.

Due to the low awareness and knowledge of brucellosis, the health of occupationally exposed populations and public food safety need more attention. It has been reported that a lack of knowledge about the disease could potentially lead to a delay in seeking medical support and, hence, a delay in the diagnosis and treatment of the disease [104,105]. Misdiagnosis often leads to a delay in treatment and can result in long-term complications from the disease [106]. In addition, the low brucellosis awareness and knowledge level of people involved in the livestock value chain could lead to a neglect in disease prevention and incorrect practices in handling, cooking and preserving animal-based food, which poses a great threat to public food safety [97]. Knowing the high-risk behaviors associated with brucellosis infections can also promote individuals to take protective measures, such as avoiding the consumption of raw milk and uncooked meat and wearing gloves when delivering or handling abortion materials.

Many factors are thought to be related to the level of awareness and knowledge of brucellosis. Several studies in the meta-analysis have indicated that education is positively associated with awareness and knowledge levels [28, 29, 39, 62, 80, 81, 92, 93, 95, 96]. It has been shown that previous experience with brucellosis in livestock and brucellosis prevalence levels are positively correlated with awareness and knowledge levels of brucellosis [107]. A study in southwestern Ethiopia [108] suggested that the lack of awareness of zoonotic diseases in the study area might have been due to the lack of awareness-creating activities provided by public health agencies and veterinary departments in the region. In summary, a low level of awareness could be due to remoteness, a lack of health facilities, poor extension services, little training on the rearing and handling of animals, a lack of health education programs and low literacy rates, which have been reported as major contributors to the low level of awareness among dairy farmers [109]. Currently, cross-sectoral and disciplinary cooperation in the control of zoonoses is encouraged by the “One Health” framework [110,111]. Communication and cooperation between the animal and human health sectors, the agricultural sector, the education sectors, animal producers and other relevant occupational groups are very important to improve the awareness and control of brucellosis.

In the present study, greater brucellosis awareness and knowledge were reported in the respondents involved in both bovine and small ruminant production, and the awareness and knowledge level in the respondents involved in small ruminant production was higher than that in people involved in only bovine animal production. This might be because brucellosis seropositivity was higher in goats than in other species [112].

Health workers play an important role in health education and disease knowledge advocacy for occupational groups. In this study, the greatest awareness was reported in health care providers, including both animal and human health workers. This can be explained by their medical background and the training and experience they receive over their career, which proves the importance of education and training to improve the awareness of brucellosis in high-risk groups [113,114].

The results showed that the main brucellosis information sources were friends and neighbors. A low proportion of participants mentioned mass media (radio/TV) as a source of information about brucellosis; this fact may suggest that the role of television/radio as a mass media outlet for the dissemination of knowledge about brucellosis has not received much attention. This should be considered in the development of education programs regarding brucellosis control.

The strength of our meta-analysis was that the evaluation of recent studies on about brucellosis awareness and knowledge among high-risk populations, health workers, general residents and students worldwide offered the evidence-based guidance for the implementation of education services and brucellosis control measures. However, there were several limitations in this study. Obvious heterogeneity existed in the meta-analysis. Although a theoretical framework was designed for this study, it was difficult to ensure that a reasonable design and rigorous questionnaire and sampling methods were used in all original studies to complete the investigations.

In summary, mainly in Asia and Africa, an insufficient proportion of the populations in rural communities is aware of brucellosis and a low knowledge level of brucellosis was observed. Since the occupationally exposed population's perception of brucellosis influences the development and implementation of disease control strategies as well as the adoption of best practices and habits during work and life, it is very important to raise the awareness level of brucellosis in occupationally exposed populations.

## Supporting information

S1 Appendix PRISMA checklist. (DOC)

S2 Appendix Studies search strategies in the meta-analysis. (DOCX)

S3 Appendix Risk of bias assessment. (XLSX)

S4 Appendix Pooled forest and funnel plots of meta-analysis. (DOCX)

S5 Appendix Data for meta-analysis. (XLSX)
