# Supplementary material for: Brucellosis awareness and knowledge in communities worldwide: A systematic review and meta-analysis of 79 observational studies
Source: PLoS Negl Trop Dis. 2019 May 2;13(5):e0007366. doi: 10.1371/journal.pntd.0007366 (PMC6497230; doi:10.1371/journal.pntd.0007366)
Supplement: S2 Appendix — (DOCX) [file pntd.0007366.s002.DOCX]

**Search strategy of literature search:**

**PubMed：**

1. ((((brucellosis) OR brucella)) AND (((((knowledge) OR awareness) OR perception) OR KAP) OR attitude)) AND ("2010"[Publication Date] : "2018"[Publication Date])
2. ((((((zoonotic disease) OR zoonosis) OR zoonoses)) AND ((brucellosis) OR brucella)) AND (((((knowledge) OR awareness) OR perception) OR KAP) OR attitude)) AND ("2010"[Publication Date] : "2018"[Publication Date])

**Web of Science:**

1. TS: ((brucellosis OR brucella) AND (knowledge OR awareness OR perception OR KAP OR attitude))
2. TS: ((zoonotic disease OR zoonosis OR zoonoses) AND (knowledge OR awareness OR perception OR KAP) AND (brucellosis OR brucella))

**Yahoo:**

1. (brucellosis OR brucella) AND (knowledge OR awareness OR perception OR KAP OR attitude)
2. (zoonotic disease OR zoonosis OR zoonoses) AND (knowledge OR awareness OR perception OR KAP) AND (brucellosis OR brucella)

**China Nat****ional Knowledge Infrastructure:**

1. (brucella[SU] OR brucellosis[SU]) AND (knowledge[SU] OR perception[SU])

**Wan Fang:**

1. (brucella[Title/Abstract] OR brucellosis[Title/Abstract]) AND(knowledge[Title/Abstract] OR perception[Title/Abstract]) OR awareness OR attitude
