# Supplementary material for: Brucellosis awareness and knowledge in communities worldwide: A systematic review and meta-analysis of 79 observational studies
Source: PLoS Negl Trop Dis. 2019 May 2;13(5):e0007366. doi: 10.1371/journal.pntd.0007366 (PMC6497230; doi:10.1371/journal.pntd.0007366)
Supplement: S4 Appendix — (DOCX) [file pntd.0007366.s004.docx]

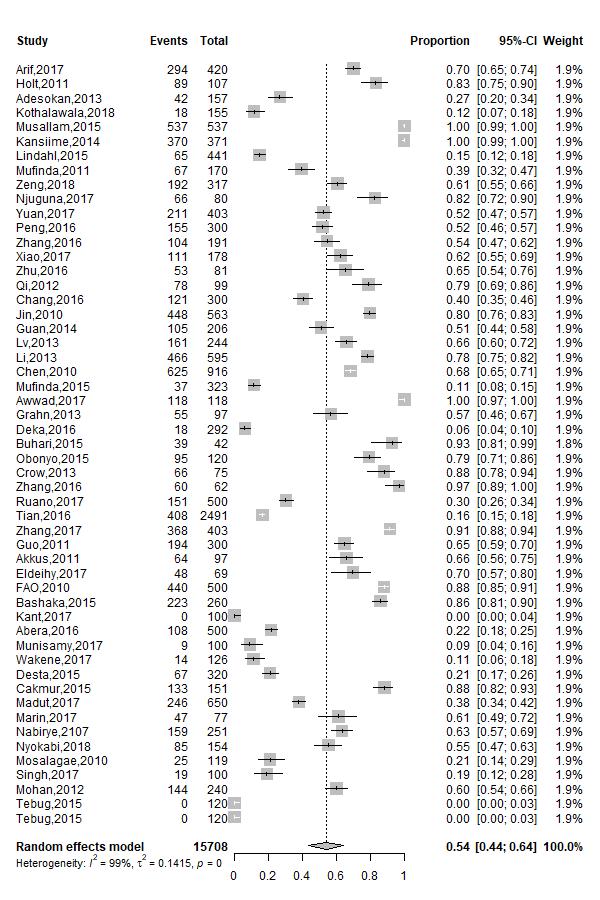


**Figure 2A. Forest plots of brucellosis awareness**


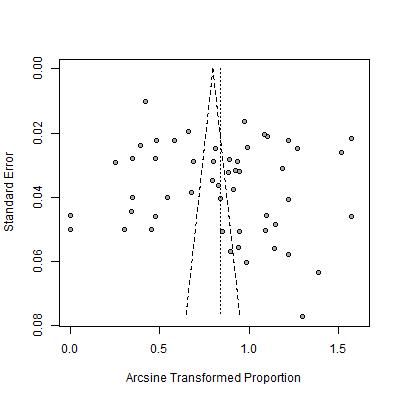


**Figure 2B. Funnel plot of brucellosis awareness.**


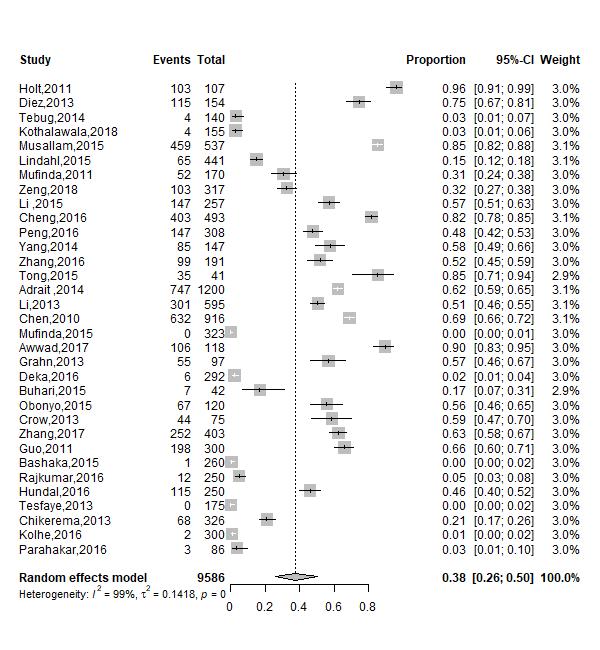


**Figure 3A. Forest plots of brucellosis knowledge of its zoonotic nature.**


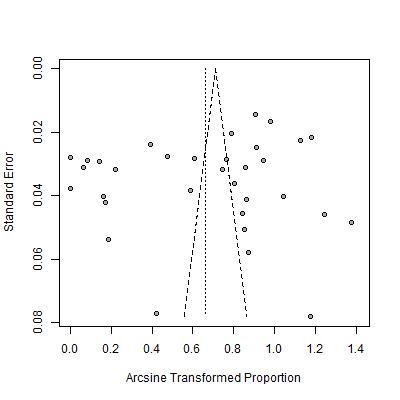


**Figure 3B. Funnel plot of brucellosis knowledge of its zoonotic nature**


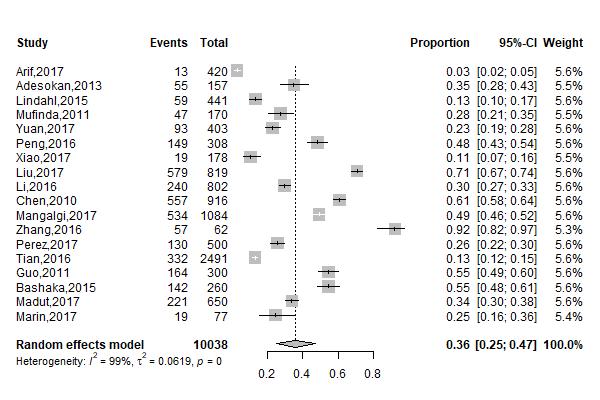


**Figure 4A. Forest plots of brucellosis knowledge regarding the mode of transmission.**


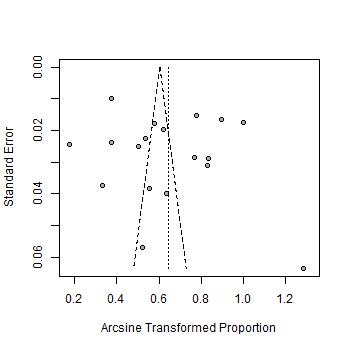


**Figure 4B. Funnel plots of brucellosis knowledge regarding the mode of transmission.**


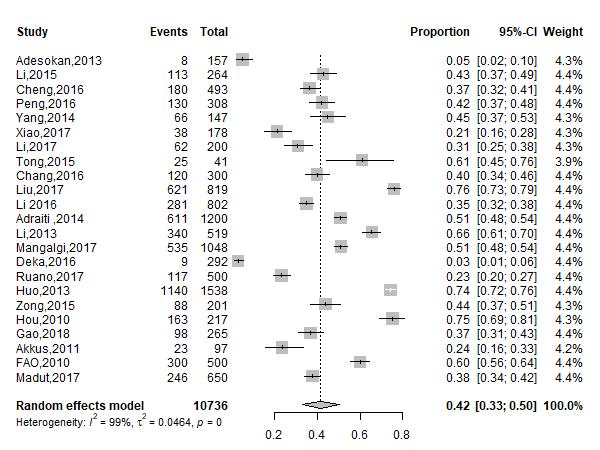
 **Figure 5A. Forest plots of knowledge regarding symptoms of human brucellosis**


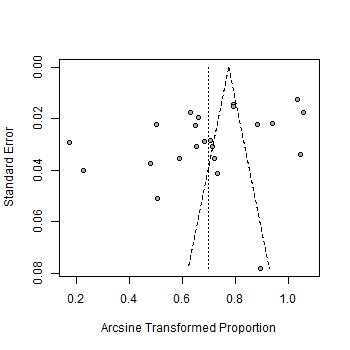


**Figure 5B. Forest plots of knowledge regarding symptoms of human brucellosis**


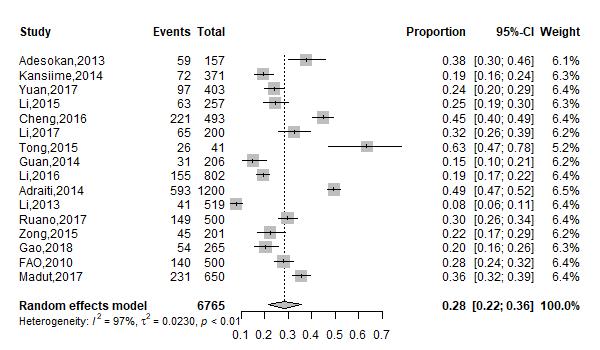
 **Figure 6A. Forest plots of knowledge regarding signs of animal brucellosis.**


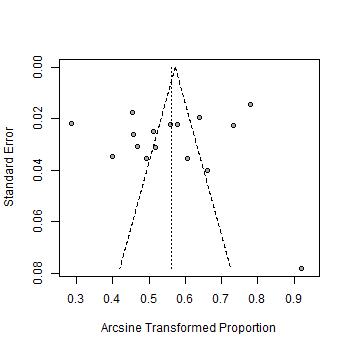


**Figure 6B. Funnel plots of knowledge regarding signs of animal brucellosis.**


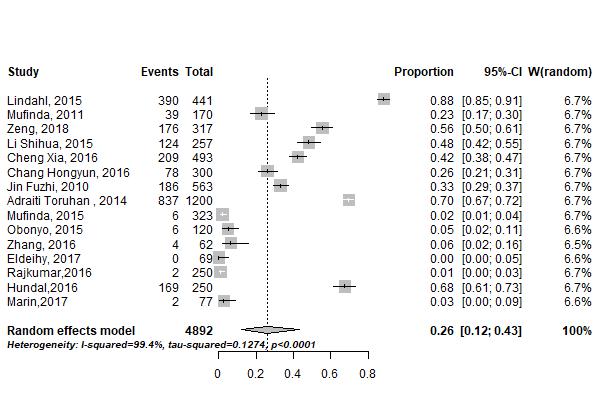


**Figure 7A. Forest plots of knowledge regarding vaccination as a preventive measure.**


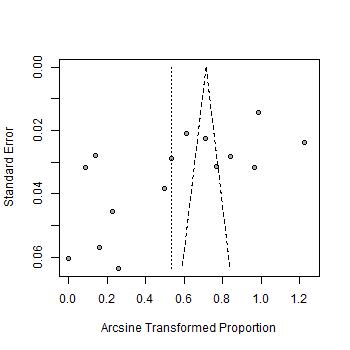


**Figure 7B. Funnel plot of knowledge regarding vaccination as a preventive measure.**
